# Survey of VA Laboratory Practices for Carbapenem-resistant Acinetobacter baumannii and Pseudomonas aeruginosa

**DOI:** 10.1017/ash.2024.212

**Published:** 2024-09-16

**Authors:** Aubrey Sawyer, Cara Ray, James Stacey Klutts, Margaret Fitzpatrick, Katie Suda, Natalie Hicks, Martin Evans, Makoto Jones, Christopher Pfeiffer, Charlesnika Evans

**Affiliations:** Northwestern University, Feinberg School of Medicine; Department of Veterans Affairs; University of Colorado Anschutz Medical Center; University of Pittsburgh School of Medicine; National Infectious Diseases Service (NIDS); Vha

## Abstract

**Background:** Carbapenem-resistant Acinetobacter baumannii (CRAB) and Pseudomonas aeruginosa (CRPA) are drug-resistant pathogens causing high mortality rates with limited treatment options. Understanding the incidence of these organisms and laboratory knowledge of testing protocols is important for controlling their spread in healthcare settings. This project assessed how often Veterans Affairs (VA) healthcare facilities identify CRAB and CRPA and testing practices used. **Method:** An electronic survey was distributed to 126 VA acute care facilities September-October 2023. The survey focused on CRAB and CRPA incidence, testing and identification, and availability of testing resources. Responses were analyzed by complexity of patients treated at VA facilities (High, Medium, Low) using Fisher’s exact tests. **Result:** 77 (61.1%) facilities responded, most in urban settings (85.4%). Most respondents were lead or supervisory laboratory technologists (84.2%) from high complexity facilities (69.0%). Few facilities detected CRAB ≥ once/month (4.4%), with most reporting that they have not seen CRAB at their facility (55.0%). CRPA was detected more frequently: 19% of facilities with isolates ≥ once/month, 29.2% a few times per year, and 26.9% reporting had not seen the organism. No differences in CRAB or CRPA incidence was found by facility complexity. Nearly all facilities, regardless of complexity, utilize the recommended methods of MIC or disk diffusion to identify CRAB or CRPA (91.9%) with remaining facilities reporting that testing is done off-site (7.8%). More high complexity facilities perform on-site testing compared to low complexity facilities (32.0% vs 2.7%, p=0.04). 83% of laboratories test for Carbapenemase production, with one-fourth using off-site reference labs. One-fourth of facilities perform additional antibiotic susceptibility testing for CRAB and CRPA isolates, most of which test for susceptibility to combination antibiotics; no differences between complexities were found. Agreement that sufficient laboratory and equipment resources were available was higher in high complexity than in medium complexity facilities (70.7% vs 33.3%, p=0.01), but not low complexity facilities (43.8%). **Conclusion:** Having timely and accurate testing protocols for CRAB and CRPA are important to quickly control spread and reduce associated mortality. This study shows that most VA protocols follow recommended testing and identification guidelines. Interestingly, there was no difference in CRAB or CRPA incidence for facilities providing higher vs lower complexity of care. While high and low complexity facilities generally reported sufficient resources for CRAB and CRPA evaluation, some medium-complexity labs, who may feel more compelled than low-complexity labs to bring testing in house, reported that additional resources would be required.

